# A Manganin Thin Film Ultra-High Pressure Sensor for Microscale Detonation Pressure Measurement

**DOI:** 10.3390/s18030736

**Published:** 2018-03-01

**Authors:** Guodong Zhang, Yulong Zhao, Yun Zhao, Xinchen Wang, Xueyong Wei, Wei Ren, Hui Li, You Zhao

**Affiliations:** 1 State Key Laboratory for Manufacturing Systems Engineering, Xi’an Jiaotong University, Xi’an 710049, China; zhangguodong@stu.xjtu.edu.cn (G.Z.); zhaoyun@stu.xjtu.edu.cn (Y.Z.); wangxinchen@stu.xjtu.edu.cn (X.W.); seanwei@mail.xjtu.edu.cn (X.W.); zhaoyou319@stu.xjtu.edu.cn (Y.Z.); 2 National Defense Key Laboratory of Pyrotechnical S&R Technology, The 213rd Research Institute of China Ordnance Industry, Xi’an 710061, China; rw0192@163.com (W.R.); lihuilingshi@163.com (H.L.)

**Keywords:** ultra-high pressure sensor, manganin thin film, MEMS technology, detonation pressure, microscale measurement

## Abstract

With the development of energetic materials (EMs) and microelectromechanical systems (MEMS) initiating explosive devices, the measurement of detonation pressure generated by EMs in the microscale has become a pressing need. This paper develops a manganin thin film ultra-high pressure sensor based on MEMS technology for measuring the output pressure from micro-detonator. A reliable coefficient is proposed for designing the sensor’s sensitive element better. The sensor employs sandwich structure: the substrate uses a 0.5 mm thick alumina ceramic, the manganin sensitive element with a size of 0.2 mm × 0.1 mm × 2 μm and copper electrodes of 2 μm thick are sputtered sequentially on the substrate, and a 25 μm thick insulating layer of polyimide is wrapped on the sensitive element. The static test shows that the piezoresistive coefficient of manganin thin film is 0.0125 GPa^−1^. The dynamic experiment indicates that the detonation pressure of micro-detonator is 12.66 GPa, and the response time of the sensor is 37 ns. In a word, the sensor developed in this study is suitable for measuring ultra-high pressure in microscale and has a shorter response time than that of foil-like manganin gauges. Simultaneously, this study could be beneficial to research on ultra-high-pressure sensors with smaller size.

## 1. Introduction

With the development of microelectromechanical systems (MEMS) technology, miniaturization and integration of EMs and electronic devices are the inevitable trend. Especially, MEMS initiating explosive devices have been one of the most active fields. In order to quantitatively describe the output performance of MEMS initiating explosive devices, the measurement of ultra-high pressure in microscale has become an urgent research subject.

Generally, the detonation pressure of energetic materials (EMs) belongs to ultra-high pressure up to GPa. In the literature there are many papers proposing various ultra-high pressure sensors based on different principles for measuring ultra-high pressure [1,2,3,4,5,6,7]. Among of them, polyvinylidene fluoride (PVDF) piezoelectric sensors and manganin piezoresistive sensors are used widely in ultra-high pressure measurement. However, PVDF piezoelectric sensors cannot accurately measure the detonation pressure of EMs because they easily suffer from the influence of temperature [8]. Therefore, the manganin piezoresistive effect is employed in this study to propose a novel ultra-high pressure sensor for microscale detonation pressure measurement. 

Since the 1960s, there have been several investigations of the effect of dynamic pressure on the resistance of manganin, an alloy of nominally 84% Cu, 12% Mn, and 4% Ni [9,10]. The manganin sensor is widely used in many applications for measuring ultra-high pressure because of its high sensitivity, fast response, good linearity and low temperature coefficient of resistance [11]. Bernstein [12] introduced a manganin wire of 0.075 mm in diameter casted into an epoxy insulator. The change in resistance of the manganin wire has been measured as a function of peak shock-wave pressure up to 19 GPa. Lyle [13] presented a transducer that was made of manganin wire 0.025 mm in diameter and 7 mm long, with an electrical resistance of about 7 Ω. He reported the results of an investigation of the dynamic piezoresistive coefficient in the range of pressure from 7.7 GPa to 39.2 GPa. Huang [14] explored an H type foil-like manganin gauge, the sensitive element of which is approximately 0.254 mm long, 0.127 mm wide and 10 μm thickness. He found that the H type manganin gauge can measure the detonation pressure of small diameter explosive charge. Du [11] investigated a new type of manganin gauge prepared by thin film technique. This manganin gauge has a sensitive element of 5.4 mm long, 1 mm wide and 2 μm thickness. Du [15] suggested that the thin film manganin gauge has a fast response in impact loading experiment by two-stage light-gas gun. 

However, few studies have been done on ultra-high pressure measurement in microscale. The existing manganin sensors have two shortcomings: first, as the geometries of most detonation waves yielded by EMs are two-dimensional or three-dimensional rather than one-dimensional plane [16], the large sensitive element cannot accurately measure the pressure at the center; second, the thick sensitive element extends the response time of the sensor. Solving these two problems simultaneously is very important and favorable for measuring the detonation pressure of EMs in microscale.

The aim of the present paper is to develop a manganin thin film ultra-high pressure sensor based on MEMS technology for measurement of ultra-high pressure produced by EMs in microscale. The small sensitive element is suitable for microscale measurement and the thin sensitive element reduces the response time of the sensor. The results reported here may be of interest to the study of ultra-high pressure sensors with faster response and smaller size.

## 2. Experimental Section

### 2.1. Design Principle

**Figure 1:**
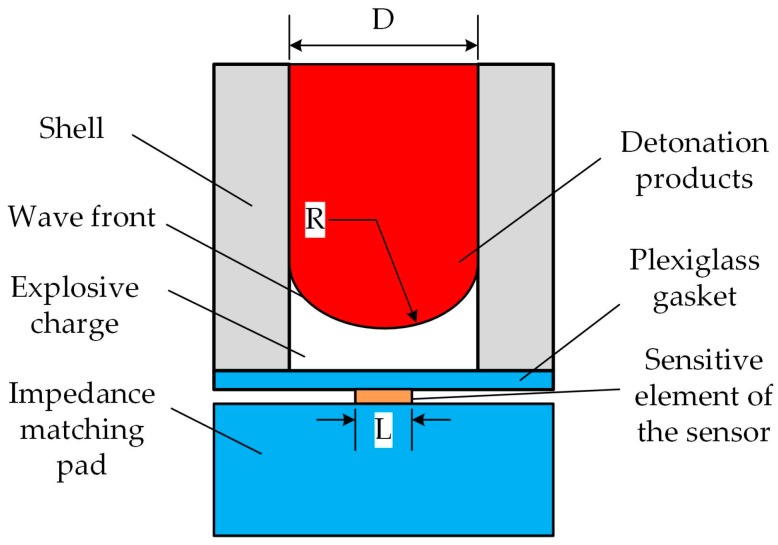
Schematic diagram of the sensor arrangement.

The purpose of this study is to develop a manganin thin film ultra-high pressure sensor based on MEMS technology for measurement of the pressure explosively generated by EMs in microscale. Generally, the dimension of microscale charge is from 0.5 mm to 5 mm in diameter. In these cases, the bending effect of detonation wave fronts is enhanced, thus making detonation wave fronts become two-dimensional convex spherical ones. Specifically, as shown in Figure 1, the pressure on the wave front is maximum at the center, and it attenuates along the radial direction and has an axial symmetry distribution. If the size of the sensor’s sensitive element is much smaller than the radius of curvature of the wave front, this shock wave is similar to a plane wave for the sensitive element, which is relatively ideal for the measurement. In practice, the following empirical formula generally needs to be met [14]:

(1) $$\frac{L}{R}\leq \frac{1}{5}$$

where *L* is structure size of the sensitive element, *R* denotes the radius of curvature of the wave front. For most condensed explosives, the maximum radius of curvature of the detonation wave is about 2~3.5 times the diameter of the charge [17]. Then, we can derive the following inequation:

(2) L≤(0.4∼0.7)D

where *D* is the diameter of the charge. In order to design the sensitive element expediently, we take the form

(3) L≤0.4D

For example, if we want to measure accurately the detonation pressure of the charge with 3 mm in diameter, the structure size of the sensitive element must not exceed 1.2 mm. 

However, the proportional relationship between maximum radius of curvature of the detonation wave and diameter of the charge is uncertain for different condensed explosives, especially the new explosive. In addition, for the measurement of pressure at the center of detonation, the smaller size of the sensitive element is better. But a lower limit of the dimension of the sensitive element is decided by processing conditions. Therefore, it is not reasonable that we choose the upper size of the sensitive element derived from Equation (3). In order to ensure the reliability and accuracy of the measurement, we propose a parameter called reliable coefficient which can be determine by processing conditions and experiences. In Equation (3) can be recast into the following equality because of the reliable coefficient

(4) L=0.4kD

where *k* is reliable coefficient within the range from 0 to 1. That is to say, Inequation (1) is a recognized empirical criterion for designing sensitive elements, and reliable coefficient reflects the designer’s influence factors on the design of sensitive elements. We give a definite design criterion by combining these two aspects, as shown in Equation (4). Given that *k* = 1/6 based on our processing conditions in this study, we obtain that the maximum size of the sensitive element is 0.2 mm which is ideal for measuring accurately the detonation pressure of the charge with diameter of 3 mm or more.

Generally, detonation pressure of microscale charge is approximately ten GPa. In such condition, the sensitive element adopts a low resistance for three reasons [16]: first, it is not necessary to use high resistance under high pressure; second, a low resistance can effectively reduce the area of the sensitive element to adapt microscale measurement; third, the piezoresistive effect of four leads can be ignored if the resistance of the sensitive element is much smaller than the characteristic impedance of transmission cables. In present paper, we set the resistance of the sensitive element equal to 0.5 Ω. If the resistivity of plating manganin is 50 μΩ∙cm [18] and the length of the sensitive element is 0.2 mm, then we have the following equation:

(5) w⋅h=0.2mm⋅μm

where *w* and *h* are width and thickness of the sensitive element, respectively. If *h* = 2 μm, we can derive that *w* = 0.1 mm. 

In a word, in order to measure accurately the detonation pressure of the charge with diameter of 3 mm or more, we designed a sensitive element with 0.2 mm long, 0.1 mm wide and 2 μm thickness in theory.

### 2.2. Sensor Design and Fabrication

**Figure 2:**
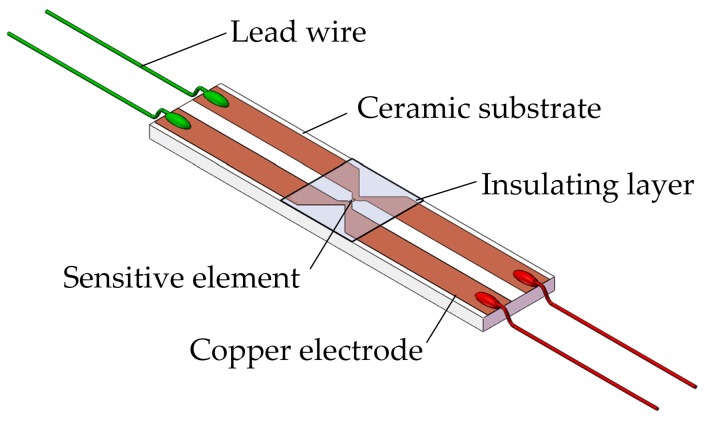
Diagrammatic sketch of the designed sensor.

A manganin thin film ultra-high pressure sensor based on MEMS technology for measuring the detonation pressure of microscale charge was designed as depicted in Figure 2. The sensor employs a sandwich structure: 96% alumina ceramics of 0.5 mm in thickness was used as the substrate because of its good adhesion with manganin thin film, chemical corrosion resistance and excellent electrical insulation under high pressure; the sensitive element was made of manganin alloy with a straight strip type, two copper electrodes were extracted at each end of the sensitive element; the insulating layer adopted polyimide of 25 μm in thickness whose impedance matches with that of explosives [19]. 

**Figure 3:**
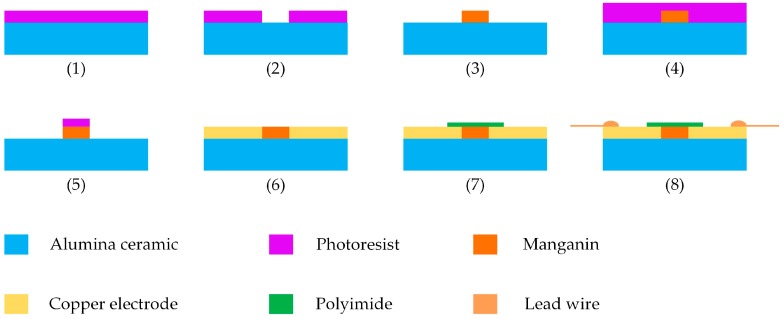
Fabrication process of the designed sensor.

**Figure 4:**
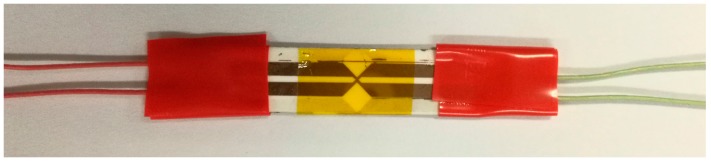
Photograph of the fabricated sensor.

In order to realize the miniaturization of the sensitive element, MEMS technology was employed. Specific fabrication process is illustrated in Figure 3: (1) photoresist was coated on the clean substrate by using a coater; (2) the pattern of the sensitive element on the mask plate was transferred to the substrate by lithography and development; (3) the manganin sensitive element was formed by magnetron sputtering, and then the heat treatment of 300 ℃ for one hour was carried out to increase piezoresistance coefficient of the manganin sensitive element; (4)−(6) the same procedures were implemented to form copper electrodes; (7) the polyimide was affixed to the sensitive element; (8) the lead wires were welded to the ends of four copper electrodes. The physical diagram of the sensor is shown in Figure 4.

A standard four-probe method was applied to reduce the influence of lead resistance and contact resistance and to improve signal-to-noise-ratio (SNR) of the sensor. Two lead wires with the same color were connected with constant current source as the input terminal, the others were attached to the oscilloscope as the output.

### 2.3. Piezoresistance Coefficient of the Sensor

Manganin has been extensively studied in the last few decades. Many researches indicate that there is an empirical relation between resistance change of manganin and stress [20],

(6) $$\frac{\Delta R}{R_{0}}=K_{P}\cdot P$$

where Δ*R* is the resistance change, *R*_0_ is the initial resistance, *P* is the stress component in the shock direction and *K_p_* denotes piezoresistance coefficient that is a function of material composition and installation conditions [16]. In general, the static piezoresistance coefficient will not have the same value as the dynamic piezoresistance coefficient for the same material. In addition, the dynamic piezoresistance coefficients obtained in different experimental arrangements may not agree, depending on the time duration of the measurement in relation to the shock equilibrium time across the specimens’ dimensions [9]. Therefore, in order to obtain more accurate measurement results, the manganin sensor needs dynamic calibration in the same experimental arrangements as the practical applications. 

However, calibration of the manganin sensor through shock experiments is a costly procedure. It would be advantageous if *K_p_* could be obtained from static high-pressure experiments. Rosenberg [21] has shown that if the ratio of thickness to diameter for a thin layer is less than 0.5% one can consider the layer as though it were subjected to purely uniaxial strain. This strain state also exists in shocked specimens undergoing planer shock loading. Under this circumstance, the static and dynamic calibration curves agree within 2%. In this study, the accuracy of the sensor is designed to be approximately 4%. According to the above design principle, it can be considered that the sensitive element is subjected to one-dimensional shock wave. Therefore, the steel anvil cells configuration with a manganin thin film (aspect ratio of 0.5% or less) can be used as the static analog to the uniaxial strain dynamic experiments. 

**Figure 5:**
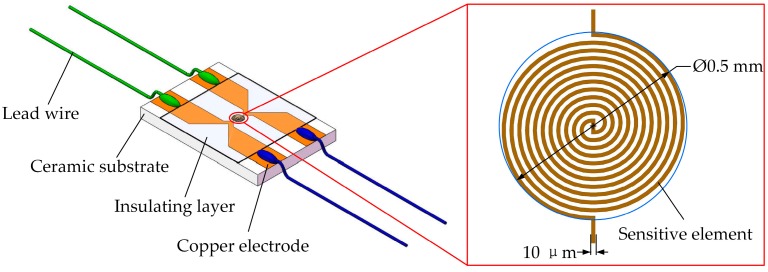
The sensor for measuring piezoresistance coefficient.

**Figure 6:**
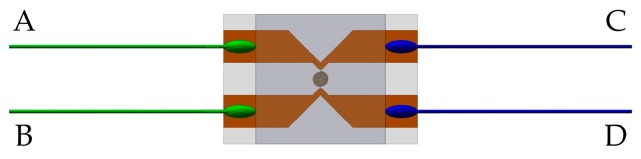
Standard four-terminal resistance measurement technique.

It can be seen from Equation (6) that piezoresistance coefficient is relative change rate of resistance of the sensitive element under pressure. In order to obtain well measurable resistance changes under small pressure, a larger initial resistance of the sensitive element is better. From the microcosmic viewpoint, the relative change rate of resistance of manganin is related to the Fermi energy of electrons and the spin of manganese ions [9]. In other words, piezoresistance coefficients of the same batch of manganin sensitive elements are consistent in theory no matter what their shapes look like. Therefore, the spiral sensitive element manufactured by MEMS technology is employed to increase the resistance, as shown in Figure 5. It is in a circle of 0.5 mm in diameter and has a minimum line width of 10 μm. Its thickness, which is identical with that of the same batch of sensors for dynamic measurement, is 2 μm. Then we can know that the aspect ratio of the spiral sensitive element is 0.4%. The resistance of the spiral sensitive element is determined by standard four-terminal resistance measurement technique. As shown in Figure 6, *R_AB_*, *R_AC_*, *R_CD_* and *R_BD_* were measured by using a digital multimeter (type FLUKE 8845A). Thus, we can figure out the resistance of the spiral sensitive element through the following equation:

(7) $$R_{g}=\left(R_{AB}+R_{CD}-R_{AC}-R_{BD}\right)/2$$

**Figure 7:**
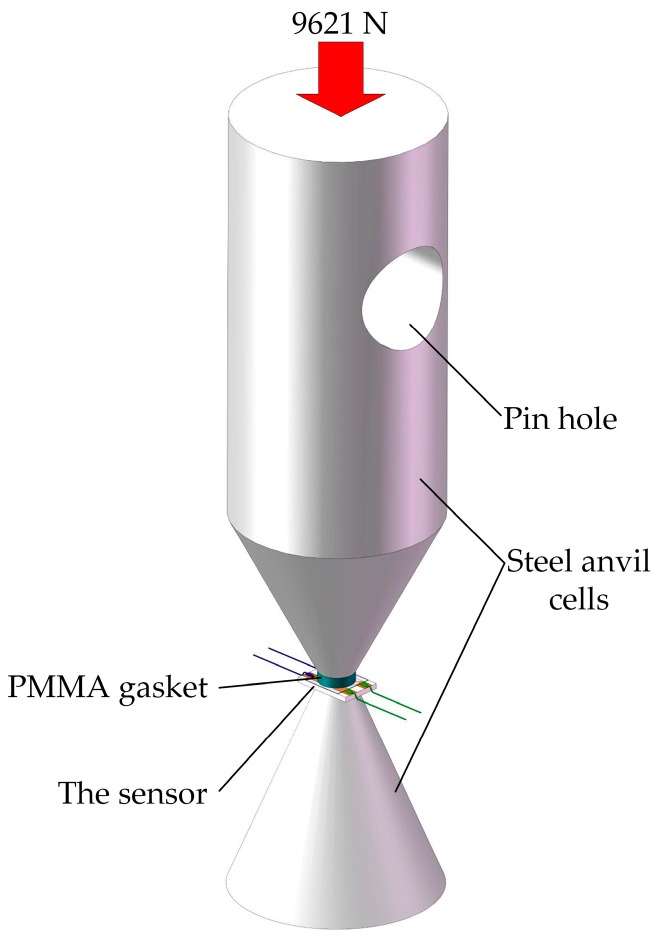
Steel anvil cells configuration.

The whole sensor for measuring piezoresistance coefficient has a size of about 6 × 4 × 0.5 mm^3^. As Figure 7 shows, it was inserted between two steel anvil cells using a 1 mm thick polymethyl methacrylate (PMMA) gasket to insulate the sensor from the steel anvil, which ensures the same installation conditions as dynamic test. An electro-mechanical universal testing machine, which could apply standard quasi-static force for a certain time, was used to compress the whole assembly. The top steel anvil cell and the electro-mechanical universal testing machine were installed together by a pin hole and a pin shaft. Given that the diameter of anvil face is 3.5 mm, we can know that a force of 9621 N applied to the top steel anvil cell corresponds to a pressure of 1 GPa acting on the sensitive element by dividing the force by the area of anvil face. The measurements of resistance changes were taken with a digital multimeter (type FLUKE 8845A). During the test, five sensors in the same batch were used for reducing the random errors. Their test results are listed in Table 1. Then we can calculate that the piezoresistive coefficient of manganin thin film ultra-high pressure sensor fabricated in this study is 0.0125 GPa^−1^ by substituting the average value in Equation (6). 

### 2.4. Sensor Application in Dynamic Test

In this section, the sensor shown in Figure 4 is applied to measure the detonation pressure exerted by micro-detonator with a charge diameter of 3.42 mm. 

**Figure 8:**
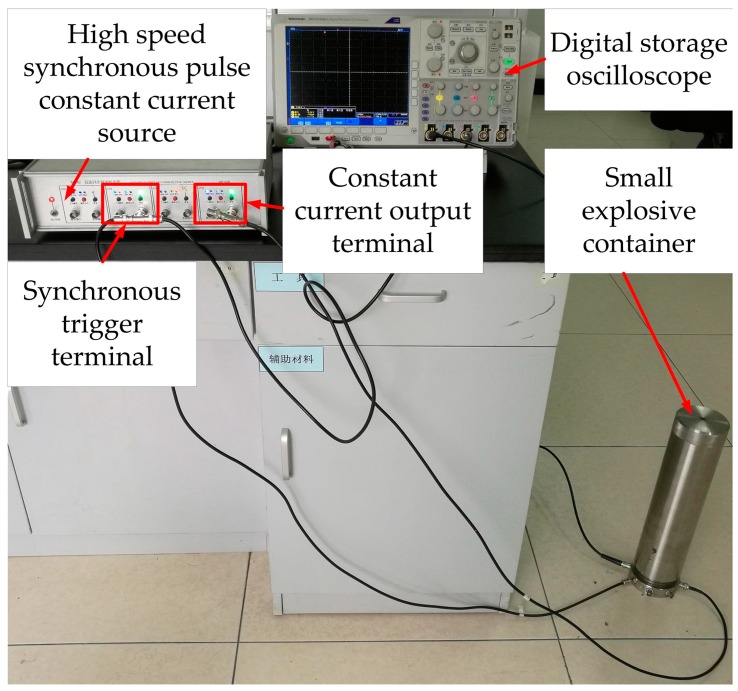
Dynamic testing system.

**Figure 9:**
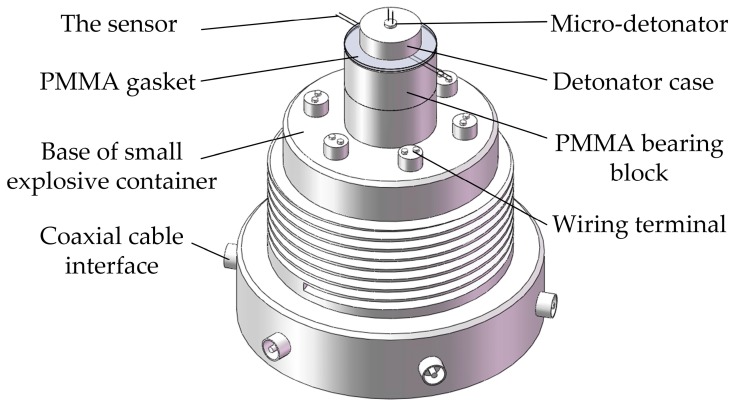
Internal structure diagram of small explosive container.

The dynamic testing system includes digital storage oscilloscope (type TDS-2014B), high speed synchronous pulse constant current source (type MH4E) and small explosive container, as illustrated in Figure 8. In the small explosive container, as shown in Figure 9, the PMMA bearing block was pasted on the base of small explosive container with cyanoacrylate glue; the sensor was inserted between the PMMA gasket and the PMMA bearing block using epoxy resin adhesive; the micro-detonator was placed above the PMMA gasket through the case; the center of PMMA bearing block, sensitive element of the sensor, PMMA gasket and micro-detonator were consciously aligned to ensure the measurement accuracy. The input end of the sensor was welded with a wiring terminal, and the corresponding coaxial cable interface was connected with constant current output terminal of the constant current source through a coaxial cable. The same method was used to attach the output end of the sensor to the digital storage oscilloscope. Similarly, the fuses of micro-detonator were linked to synchronous trigger terminal of the constant current source. The shell and base of small explosive container were in threaded connection.

In order to ensure the safety and reliability of the dynamic test, debugging the test system is necessary. First, make a short circuit of the fuses of micro-detonator and open the oscilloscope and constant current source for about ten minutes. Second, check whether the constant current source has a normal current output. Third, set up the transverse ordinate and the trigger level of the digital storage oscilloscope for ensuring that the voltage signals can be captured. 

When the test system was in good condition, initiation circuit of micro-detonator was connected. Then, the synchronous trigger terminal of the constant current source triggered the detonator and the digital storage oscilloscope synchronously through coaxial cables. Constant current output terminal of the constant current source provided a constant current to the input end of the sensor. The output signals of the sensor were recorded in the oscilloscope. The detonation wave produced by micro-detonator exerted on the sensitive element, which caused the change of resistance of the sensitive element. Correspondingly, the voltage signals of the oscilloscope changed. We can calculate the detonation pressure by the relative change rate of the voltage.

## 3. Results and Discussion

### 3.1. Calculation Method of Explosion Pressure

**Figure 10:**
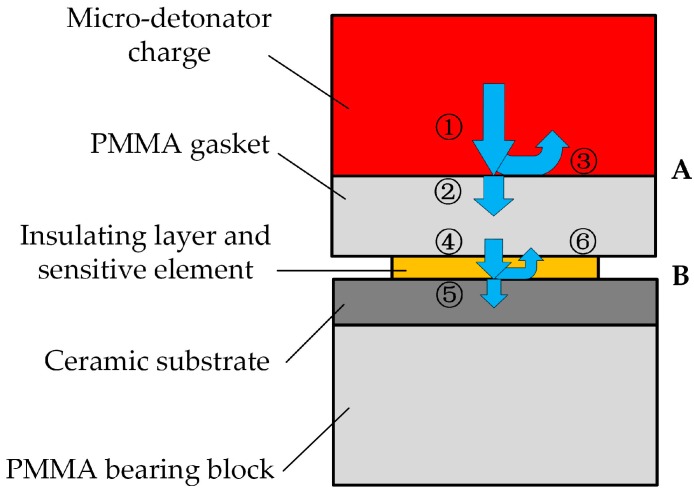
Propagation model of the detonation wave.

The reflection of detonation wave is shaped when it propagates between different materials. As Figure 10 shows, there are two reflection interfaces from the generation of detonation wave to the resistance changes of the sensitive element caused by detonation wave. It should be noted that the insulating layer and sensitive element can be ignored because of their micron-level thickness. “A” stands for the interface between micro-detonator charge and PMMA gasket, and “B” represents the interface between PMMA gasket and ceramic substrate. In order to calculate the detonation pressure accurately, these reflections must be considered. The numbers in the diagram are the corresponding incident wave, transmitted wave and reflected wave and they will appear as subscripts in the process of calculation. 

Theoretically, we consider that the transmitted wave passing through the sensitive element will cause the resistance changes of the sensitive element. Therefore, according to Equation (6) and the condition of power supply with the constant current source, we obtain the following equation

(8) $$P_{5}=\frac{1}{K_{P}}\cdot \frac{\Delta V}{V_{0}}$$

where *P*_5_ is the pressure produced by transmitted wave on the interface B and the subscript corresponds to the number in Figure 10; *K_P_* is piezoresistance coefficient of the sensor; Δ*V* and *V*_0_ denote voltage variation and initial voltage displayed in the oscilloscope, respectively. 

As for the reflection on the interface B, we have the formula of impact impedance matching according to conservation of mass and momentum on both sides of the wave front [17]

(9) $$\frac{P_{5}}{P_{4}}=\frac{2{\left(\rho_{0}C_{0}\right)}_{CE}}{{\left(\rho_{0}C_{0}\right)}_{PMMA}+{\left(\rho_{0}C_{0}\right)}_{CE}}$$

where *P*_4_ is the pressure produced by incident wave on the interface B, (*ρ*_0_*C*_0_)_CE_ and (*ρ*_0_*C*_0_)_PMMA_ are impact impedance of 96% alumina ceramics and PMMA, respectively. Similarly, the formula of impact impedance matching of micro-detonator charge and PMMA gasket on the interface A can be presented in the form

(10) $$\frac{P_{1}}{P_{2}}=\frac{{\left(\rho_{0}C_{0}\right)}_{PMMA}+{\left(\rho_{0}C_{0}\right)}_{EX}}{2{\left(\rho_{0}C_{0}\right)}_{PMMA}}$$

where *P*_1_ is detonation pressure, *P*_2_ is the pressure produced by transmitted wave on the interface A, (*ρ*_0_*C*_0_)_EX_ is impact impedance of explosive charge of micro-detonator. The main component of micro-detonator is CL-20 whose charge density is 1.8 g/cm^3^ and steady detonation velocity is 8.5 mm/μs.

Based on the momentum conservation equation and the relationship between shock wave velocity and particle velocity, initial velocity of shock wave in materials can be expressed as

(11) $$C_{0}=\frac{c+\sqrt{c^{2}+4\lambda \frac{P}{\rho }}}{2}$$

where *c* and *λ* are characteristic constants of materials, *P* is the pressure produced by transmitted shock wave, *ρ* is density of materials. From this equation, initial velocity of shock wave in PMMA and 96% alumina ceramics can be written as

(12) $$C_{0-PMMA}=\frac{c_{1}+\sqrt{c_{1}^{2}+4\lambda_{1}\frac{P_{2}}{\rho_{1}}}}{2}$$

(13) $$C_{0-CE}=\frac{c_{2}+\sqrt{c_{2}^{2}+4\lambda_{2}\frac{P_{5}}{\rho_{2}}}}{2}$$

where we use the values of characteristic constants of PMMA *c*_1_ = 2.87 km/s and *λ*_1_ = 1.88, the density of PMMA *ρ*_1_ = 1.18 g/cm^3^, characteristic constants of 96% alumina ceramics *c*_2_ = 5.60 km/s and *λ*_2_ = 1.65, the density of 96% alumina ceramics *ρ*_2_ = 3.65 g/cm^3^. 

The detonation wave will decay in the PMMA gasket and the attenuation rule takes the form

(14) $$P_{4}=P_{2}\times e^{-0.3587x}(0\leq x\leq 5\mathrm{mm})$$

where *x* is the thickness of PMMA gasket. Given that *x* = 1 mm in this experiment, we obtain

(15) $$P_{2}=\frac{P_{4}}{0.6986}$$

On combining Equations (8)–(10), (12), (13) and (15), we can calculate the detonation pressure *P*_1_.

### 3.2. Experimental Result

**Figure 11:**
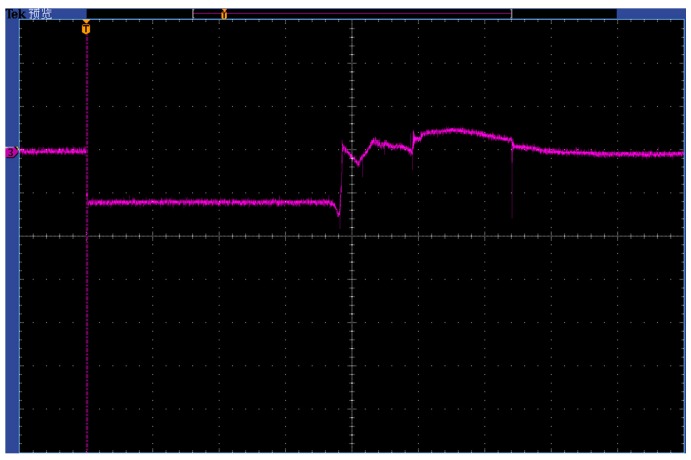
Voltage signals collected by the oscilloscope.

**Figure 12:**
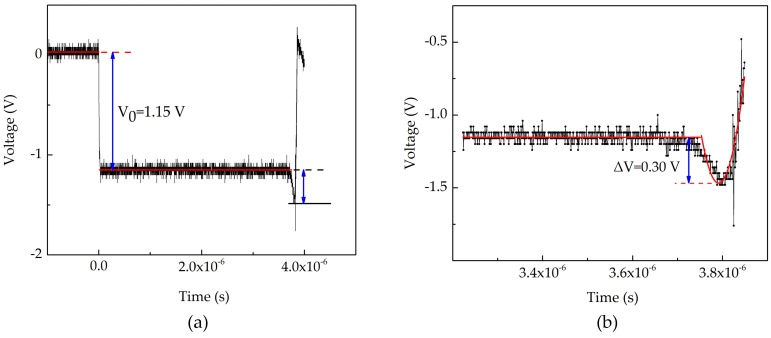
(**a**) Initial voltage; (**b**) Voltage variation.

In the process of dynamic test, voltage signals were stored in the digital storage oscilloscope, as shown in Figure 11. The first horizontal line on the left side of Figure 11 is the voltage state of the sensor without power supply. The second horizontal line denotes the voltage state of the sensor when the constant current source provides the current. The following voltage change are attributable to the resistance change of the manganin sensitive element caused by detonation wave. The other voltage states are complicated and they may be the results of the multiple reflections of detonation wave or the damage of the sensor. The data processing only needs two previous changes of the voltage and the processed results are shown in Figure 12. The red lines are the fitting curves of experimental data. From Figure 12a,b, it can be seen that the initial voltage of the sensor is 1.15 V and voltage variation of the sensor is 0.30 V. Then we have *P*_5_ = 20.8696 GPa. Upon building the simultaneous equations of Equations (9), (10), (12), (13) and (15), substituting the known parameters into them, and solving for *P*_1_. Finally, we obtain the detonation pressure of micro-detonator is 12.66 GPa. From Figure 12b, we can know that the response time of the sensor is about 37 ns which is shorter than that of ordinary foil manganin sensors [15].

### 3.3. Discussion

The true value of detonation pressure is difficult to obtain by making numerical simulation and calculation with formula. Theoretically, the traditional foil-like manganin gauges which offer a large and thick sensitive element are not suitable for measuring microscale detonation pressure, which indicates that there is no comparison between the previous measurement results and the results of this article for micro-detonator used in this study. Therefore, the total error of the measurement is usually decomposed into several aspects including principle error of measuring method, the error of measuring instruments, reading error and calculation error. In this study, the principle error of measuring method is greatly reduced by introducing the reliable coefficient and employing MEMS technology. However, the accuracy of detonation pressure determination can be further increased by using dynamic calibration experiments to obtain the piezoresistive coefficient, and it can also be improved with better instrumentations. Moreover, the measurement uncertainty should be obtained with multiple dynamic experimental data according to the ISO guide to the expression of uncertainty in measurement (GUM) in future work. 

## 4. Conclusions

This paper develops a novel manganin thin film ultra-high pressure sensor for measuring ultra-high pressure. In order to satisfy the measurement of detonation pressure of EMs in microscale and achieve short response time, MEMS technology is employed in the manufacturing process to reduce the size and thickness of the sensor. In addition, the reliable coefficient is proposed for designing the sensitive element of the sensor. The dynamic test indicates that the sensor realizes the measurement for detonation pressure of micro-detonator with a charge diameter of 3.42 mm. The response time of the sensor is about 37 ns which is shorter than that of ordinary foil-like manganin sensors. In addition, the research reported here would provide better insight to the research of ultra-high pressure sensors with faster response and smaller size. Future work will be focused on improvement of measuring accuracy.

## Figures and Tables

**Table 1:** The test results of five sensors.

Sensor Number	Resistance Change (Ω)	Initial Resistance (Ω)
1	0.32	25.14
2	0.29	25.09
3	0.33	25.18
4	0.28	24.98
5	0.35	25.23
Average value	0.314	25.124
